# A gene co-association network regulating gut microbial communities in a Duroc pig population

**DOI:** 10.1186/s40168-020-00994-8

**Published:** 2021-02-21

**Authors:** Antonio Reverter, Maria Ballester, Pamela A. Alexandre, Emilio Mármol-Sánchez, Antoni Dalmau, Raquel Quintanilla, Yuliaxis Ramayo-Caldas

**Affiliations:** 1 CSIRO Agriculture and Food, St. Lucia, Brisbane, Queensland 4067 Australia; 2 Animal Breeding and Genetics Program, IRTA, Torre Marimón, 08140 Caldes de Montbui, Barcelona, Spain; 3 Centre for Research in Agricultural Genomics (CRAG), CSIC-IRTA-UAB-UB, Universitat Autònoma de Barcelona, 08193 Bellaterra, Spain; 4 Animal Welfare Subprogram, IRTA, 17121 Monells, Girona, Spain

**Keywords:** Microbiota, Pig, Gene network, Regulators, Protist, Bacteria

## Abstract

**Background:**

Analyses of gut microbiome composition in livestock species have shown its potential to contribute to the regulation of complex phenotypes. However, little is known about the host genetic control over the gut microbial communities. In pigs, previous studies are based on classical “single-gene-single-trait” approaches and have evaluated the role of host genome controlling gut prokaryote and eukaryote communities separately.

**Results:**

In order to determine the ability of the host genome to control the diversity and composition of microbial communities in healthy pigs, we undertook genome-wide association studies (GWAS) for 39 microbial phenotypes that included 2 diversity indexes, and the relative abundance of 31 bacterial and six commensal protist genera in 390 pigs genotyped for 70 K SNPs. The GWAS results were processed through a 3-step analytical pipeline comprised of (1) association weight matrix; (2) regulatory impact factor; and (3) partial correlation and information theory. The inferred gene regulatory network comprised 3561 genes (within a 5 kb distance from a relevant SNP–*P* < 0.05) and 738,913 connections (SNP-to-SNP co-associations). Our findings highlight the complexity and polygenic nature of the pig gut microbial ecosystem. Prominent within the network were 5 regulators, *PRDM15*, *STAT1*, *ssc-mir-371*, *SOX9* and *RUNX2* which gathered 942, 607, 588, 284 and 273 connections, respectively. *PRDM15* modulates the transcription of upstream regulators of WNT and MAPK-ERK signaling to safeguard naive pluripotency and regulates the production of Th1- and Th2-type immune response. The signal transducer *STAT1* has long been associated with immune processes and was recently identified as a potential regulator of vaccine response to porcine reproductive and respiratory syndrome. The list of regulators was enriched for immune-related pathways, and the list of predicted targets includes candidate genes previously reported as associated with microbiota profile in pigs, mice and human, such as *SLIT3*, *SLC39A8*, *NOS1*, *IL1R2*, *DAB1*, *TOX3*, *SPP1*, *THSD7B*, *ELF2*, *PIANP*, *A2ML1*, and *IFNAR1*. Moreover, we show the existence of host-genetic variants jointly associated with the relative abundance of butyrate producer bacteria and host performance.

**Conclusions:**

Taken together, our results identified regulators, candidate genes, and mechanisms linked with microbiome modulation by the host. They further highlight the value of the proposed analytical pipeline to exploit pleiotropy and the crosstalk between bacteria and protists as significant contributors to host-microbiome interactions and identify genetic markers and candidate genes that can be incorporated in breeding program to improve host-performance and microbial traits.

**Video Abstract**

**Supplementary Information:**

The online version contains supplementary material available at 10.1186/s40168-020-00994-8.

## Background

The gut microbiota is a diverse ecosystem predominantly dominated by bacteria, but other microorganisms such as fungi and protists are also present. Influenced by the host immunity and environmental factors such as age, diet, and geography, the gut microbiota is known to be involved in many physiological functions as well as in disease pathogenesis (see for instance the recent reviews of Richard and Sokol, 2019 [1]; Torp Austvoll et al. 2020 [2]).

Lagging behind humans and model organisms, studies of the gut microbiome in livestock species have dramatically increased over the last decade thanks to a decreased metagenome next-generation sequencing cost. In the particular case of the pig, these studies have provided valuable information into the gut microbiota compositional changes, as well as associations between the porcine gut microbial communities and production traits [3–8]. In contrast, little is known about the host genetic control over the gut microbial communities in pigs. Previous studies based on classical “single-gene-single-trait” approaches and have independently evaluated the role of host-genetic genome controlling pig gut prokaryote [9–11] or eukaryote communities [5]. Therefore, ignoring the crosstalk between bacterial and protist communities, but also the contribution of gene-by-gene interactions shaping the pig gut microbial ecosystem. In their review, Aluthge et al. (2019) [12] identified the lack of sophisticated computational and bioinformatics approaches as the major bottleneck to look beyond compositional changes and instead understand the functional causative role of the pig gut microbiome.

To address this void, we propose an association weight matrix approach (AWM) [13, 14] to generate a gene co-association network where nodes are SNP mapped to or nearby gene coding regions, and found to be associated with the diversity and abundance of 31 bacterial and 6 commensal protist genera in the gut of 390 pigs genotyped for 70 K SNPs. In building and interpreting the network, we place emphasis on gene regulators responsible for the crosstalk between the host bacterial and protists communities.

## Methods

Animal care and experimental procedures were carried out following national and institutional guidelines for the Good Experimental Practices and were approved by the IRTA Ethical Committee.

### Animals, sample collection, and gut microbiome phenotypes

The pigs employed in this study are a subset of those reported in Ramayo-Caldas et al. (2020) [5]. In brief, we used a total of 405 weaned piglets (204 males and 201 females) distributed in seven batches. Samples were collected in a commercial farm at 60 ± 8 days of age. Fecal DNA was extracted with the DNeasy PowerSoil Kit (QIAGEN, Hilden, Germany), following manufacturer’s instructions. The 16S rRNA gene fragment was amplified using the primers V3_F357_N: 5′-CCTACGGGNGGCWGCAG-3′ and V4_R805: 5′-GACTACHVGGGTATCTAATCC-3′. Protist-specific primers F-566: 5′-CAGCAGCCGCGGTAATTCC-3′ and R-1200: 5′-CCCGTGTTGAGTCAAATTAAGC-3′ were used to amplify the 18S rRNA gene fragment. Amplicons were paired-end (2 × 250 nt) sequenced on an Illumina NovaSeq (Illumina, San Diego, CA, USA) at the University of Illinois Keck Center. Sequences were analyzed with QIIME2 [15] and processed into amplicon sequences variants (ASVs) at 99% of identity. Samples with less than 10,000 reads were excluded and ASVs present in less than three samples and representing less than 0.005% of the total counts were discarded. ASVs were classified to the lowest possible taxonomic level based on SILVA v123 database for 18S rRNA genes, and GreenGenes Database for Bacteria [16]. Bacteria and protist alpha diversity were evaluated with the Shannon index (Shannon, 1948); before the estimation of diversity indexes, samples were rarefied at 10,000 reads of depth. Finally, ASVs were aggregated at genera level, and only those genus present in more than 60% of the samples were considered in posteriors data analysis. In total, we captured 39 microbiome phenotypes that included 2 diversity indexes, and the relative abundance of 31 bacterial and 6 commensal protist genera. (Supplementary Table 1**).**

### Genotypes and genome-wide association studies (GWAS)

The Porcine 70 K GGP Porcine HD Array (Illumina, San Diego, CA) was used to genotype 390 out of 405 animals. We excluded single nucleotide polymorphisms (SNPs) with minor allele frequencies < 5%, rates of missing genotypes above 10%, as well as SNPs that did not map to the porcine reference genome (Sscrofa11.1 assembly). Then, to identify SNPs from the host genome associated with the alpha diversity as well as bacteria and protists relative abundances, a series of 39 GWAS were performed between 42,562 SNPs and the alpha diversity or the centered log ratio (clr) transformed genera abundance. For the GWAS, we used the GCTA software [17] using the following model at each SNP:

$$ {y}_{ijk}={\mathrm{sex}}_j+{b}_k+{u}_i+{s}_{li}{a}_l+{e}_{ijk} $$

where *y*_*ijk*_ corresponds to the microbiome phenotype under scrutiny of the *i*-th individual animal of sex *j* in the *k*-th batch; *sex*_*j*_ and *b*_*k*_ correspond to the systematic effects of *j-*th sex (2 levels) and *k*-th batch (7 levels), respectively; *u*_*i*_ is the random additive genetic effect of the *i*-th individual, collectively distributed as **u** ~ N(0, ${\sigma}_u^2$ **G**) where ${\sigma}_u^2$ is the additive genetic variance and **G** is the genomic relationship matrix calculated using the filtered autosomal SNPs based on the methodology of Yang et al. (2011) [17]; *s*_*li*_ is the genotype (coded as 0,1,2) for the *l*-th SNP of the *i*-th individual, and *a*_*l*_ is the allele substitution effect of the *l*-th SNP on the microbiome phenotype being analyzed. Following [18] and with equivalent original derivations from [19], FDR was calculated as

$$ \mathrm{FDR}=\frac{P\left(1-\frac{A}{T}\right)}{\left(\frac{A}{T}\right)\left(1-P\right)} $$

Where *P* is the *P* value tested, *A* is the number of SNP that were significant at the *P* value tested, and *T* is the total number of SNP tested. For further analyses and in order to allow for direct comparison across phenotypes, estimated SNP effects were standardized by dividing them by the standard deviation of all SNP effects.

### Association weight matrix, regulatory impact factors, and gene co-association networks

We used the AWM methodology [13, 14] in combination with the regulatory impact factors (RIF) algorithm [20] to identify the SNP, anchored to genes, to be included in the co-association gene network. Genes in the AWM are tagged by SNP that were found to be either associated with the key phenotype (alpha diversity), pleiotropic, or key regulators. In detail, the AWM was built in accordance with the following steps:

#### Initial search of SNP in genes (SNP-gene)

Using a population of 20 European pig breeds, Muñoz et al. (2019) [21] reported linkage disequilibrium of r2 > 0.2 for SNP pairs separated by 0.05 Mb. Therefore, we selected SNP located in the coding region or within 5 kb of an annotated gene based on the Sscrofa11.1 reference genome assembly.

#### SNP-genes associated with key phenotypes

Using the alpha diversity indexes for bacteria and protists as key phenotypes, we selected those SNP-Gene associated with alpha diversity and namely diversity-associated SNPs.

#### SNP-genes with pleiotropic potential

We captured the average number of phenotypes to which the diversity-associated SNPs were associated with, namely *N*_A_, and selected the remaining SNP-Genes associated (*P* < 0.05) to more than N_A_ phenotypes and referred to as pleiotropic SNPs.

#### SNP-genes with regulatory potential

We used the RIF algorithm [20] to identify key regulatory SNP. Details are as follows.

To undertake the RIF analysis, we considered the diversity-associated SNPs and the pleiotropic SNP as potential targets of all the SNP-Genes annotated as either transcription factors (TF) or microRNA genes (miRNA). The RIF algorithm is designed to detect loci with high regulatory potential in a set of loci, TFs, and microRNA genes in our case, while contrasting two biological conditions or groups, such as bacteria and protists in our case. The analysis makes use of two metrics: RIF1 and RIF2. While RIF1 prioritizes regulators that are consistently the most differentially co-associated with the highly associated potential target genes, RIF2 highlights regulators with the most altered abilities to predict the association of potential target genes.

To identify significant gene–gene interactions, we used the partial correlation and information theory (PCIT) algorithm [22] which calculates pairwise correlations between loci while accounting for the influence of a third locus. Unlike likelihood-based approaches, which invoke a parametric distribution (e.g., normal) assumed to hold under the null hypothesis and then a nominal *P* value (e.g., 5%) used to ascertain significance, PCIT is an information theoretic approach. Its threshold is an informative metric; in this case, the partial correlation after exploring all trios in judging the significance of a given correlation, which might then become a connection when inferring a network. It thereby tests all possible 3-way combinations in a dataset and only keeps correlations between loci if they are significant and independent of the expression of another locus, whereas no hard threshold is set for the correlation strength. The significance threshold for each combination of loci depends on the average ratio of partial to direct correlations. Gene interactions were predicted using correlation analysis of the SNP effects across pairwise rows of the AWM. Hence, the AWM-predicted gene interactions are based on significant co-association between SNP. In the network, every node represents a gene (or SNP), whereas every edge connecting two nodes represents a significant gene–gene interaction (based on SNP–SNP co-association). Finally, the Cytoscape software [23] was used to visualize the gene network and the CentiScaPe plugin [24] was used to calculate specific node centrality values and network topology parameters.

### Transcription factor binding motifs search

To further investigate the alleged role of the most relevant TFs, i.e., the PR/Set domain 15 (*PRDM15*) and the signal transducer and activator of transcription 1 (*STAT1*), a TF-binding motif search was implemented in the promoter region of the predicted target genes (i.e., genes with significant associations according to the AWM approach for *PRDM15* and *STAT1*. To this end, we established putative promoter regions 1 kb upstream from the start of the coding region of each target gene and downloaded the corresponding sequences in the Sscrofa11.1 porcine assembly according to Ensembl repositories [25], by means of the BioMart tool (https://www.ensembl.org/biomart/martview/). Transcription factor binding motifs for *PRDM15* and *STAT1* genes were retrieved from JASPAR database v.2020 [26] represented as position frequency matrices (PFMs). The FIMO algorithm [27] within MEME Suite [28] was subsequently employed for scanning individual matches between PFMs and retrieved promoter sequences. The Scrofa11.1 assembly was used for building a zero-order Markov model and integrated in FIMO calculations in order to correct for possible biases in nucleotide proportions. Motif occurrences with estimated *P* value below 10^− 4^ were considered significant and retained for further analyses.

### MicroRNA-binding sites search and structural inference

Following the rationale for detecting putative functional interactions among the set of key regulators identified by the AWM approach, we aimed at determining whether miRNA-mRNA significant co-associations found for relevant miRNA regulator genes were suggestive of an interaction between the miRNA and their co-associated mRNA transcripts. In this way, the ssc-miR-371 gene was among the top regulatory factors according to RIF metrics. The mRNA genes harboring SNPs significantly co-associated with the polymorphism within miR-371 (rs320008166, n.59 T > C) gene were retrieved and their 3′-UTR sequences according to the Sscrofa11.1 assembly annotation in Ensembl repositories [25] were downloaded by means of the BioMart tool (https://www.ensembl.org/biomart/martview/). In order to obtain a putative list of targeted mRNAs by ssc-miR-371, the seed region of the miRNA (2nd to 8th 5′ nucleotides of the mature miRNA) was reverse complemented and miRNA binding sites comprising perfect sequence matches between the seed and the retrieved 3′-UTRs (7mer-m8 sites) were assessed by using the *locate* tool from the SeqKit toolkit [29]. The potential structural consequences of rs320008166 (n.59 T > C) in the hairpin organization of ssc-miR-371 precursor transcript was assessed with the RNAfold software [30].

## Results

### Gene-tailored association between microbial traits

Table 1 lists the number of significant SNP and false discovery rate (FDR) at three nominal *P* value thresholds (*P* value < 0.05, 0.01, and 0.001) across the 39 phenotypes that were subjected to GWAS. The results highlight the trade-off that exists between significant SNP (the higher the better) and the FDR (the lower the better) as *P* values become more stringent. On average across the 39 phenotypes, the number of significant SNP (average FDR in brackets) at *P* values < 0.05, 0.01, and 0.001 was 4015.3 (FDR = 50.6%), 272.8 (FDR = 16.5%), and 87.6 (FDR = 5.8%), respectively. At the most stringent *P* value threshold of < 0.001, the highest number of significant SNP (*N* = 381; FDR = 1.1%) was obtained for the abundance of *Faecalibacterium*. On the other extreme, the lowest number of significant SNP (*N* = 25; FDR = 17.0%) was obtained for the abundance of *Catenibacterium*. Details of the genome map position and strength of the statistical association of the most significant SNP in each of the 39 phenotypes are given in Table 2. For each SNP–phenotype pair, the distance to and identity of the nearest gene is also listed in Table 2. With five instances, Chromosome 8 harbored the highest number of most associated SNP including one in the coding region of *SORCS2* (SNP rs320095924) and one in the coding region of *TRIM2* (rs329143797).

**Table 1:** Number of significant SNP (N) and false discovery rate (FDR, %) at three nominal *P* value thresholds and across the 39 phenotypes

Phenotype	*P* value < 0.05		*P* value < 0.01		*P* value < 0.001	
	*N*	FDR, %	*N*	FDR, %	*N*	FDR, %
*Anaerovibrio*	3836	53.13	355	11.90	105	4.04
*Blautia*	3803	53.64	232	18.26	88	4.82
*Bulleidia*	4114	49.18	196	21.63	77	5.51
*Butyricicoccus*	3830	53.22	255	16.60	92	4.61
*Campylobacter*	4088	49.53	271	15.62	69	6.15
*Catenibacterium*	4407	45.56	164	25.87	25	17.01
*Clostridium*	4024	50.40	211	20.09	67	6.34
*Collinsella*	3969	51.17	290	14.59	73	5.82
*Coprococcus*	4152	48.68	269	15.73	68	6.24
*Desulfovibrio*	3797	53.73	203	20.88	70	6.07
*Dorea*	4170	48.45	226	18.75	63	6.74
*Faecalibacterium*	3644	56.21	750	5.58	387	1.08
*Fibrobacter*	4032	50.29	225	18.83	66	6.43
*Gemmiger*	4077	49.68	238	17.80	43	9.88
*Lachnospira*	4192	48.17	248	17.07	64	6.64
*Lactobacillus*	3966	51.21	291	14.54	103	4.12
*Megasphaera*	4027	50.36	234	18.10	69	6.15
*Mitsuokella*	4201	48.06	211	20.09	58	7.32
*Oscillospira*	4161	48.57	198	21.41	68	6.24
*Parabacteroides*	4206	47.99	211	20.09	45	9.44
*Peptococcus*	4254	47.39	319	13.25	95	4.47
*Phascolarctobacterium*	3921	51.86	278	15.22	129	3.28
*Prevotella*	3952	51.41	267	15.85	66	6.43
*RFN20*	3946	51.50	299	14.14	101	4.20
*Roseburia*	4025	50.39	264	16.03	59	7.20
*Ruminococcus*	4015	50.53	265	15.97	97	4.37
*Sphaerochaeta*	4143	48.80	213	19.90	68	6.24
*Streptococcus*	4060	49.91	260	16.28	73	5.82
*Succinivibrio*	4071	49.76	314	13.46	99	4.28
*Sutterella*	4016	50.51	248	17.07	81	5.24
*Treponema*	3966	51.21	307	13.77	102	4.16
AlphaBACT	3960	51.30	314	13.46	89	4.77
*Entamoeba*	3849	52.92	335	12.61	117	3.62
*Hypotrichomonas*	3897	52.21	230	18.42	67	6.34
*Trichomitus*	3944	51.52	307	13.77	126	3.36
*Tetratrichomonas*	3998	50.76	266	15.91	64	6.64
*Neobalantidium*	3828	53.24	297	14.24	139	3.05
*Blastocystis*	3859	52.77	320	13.21	78	5.44
AlphaPROTO	4195	48.13	259	16.34	67	6.34

**Table 2:** Genome map position and strength of the association for most significant SNP in each of the 39 phenotypes

Phenotype	SNP	Chr	Bp	Candidate gene	Distance to gene (Bp)	Effect	*P* value
*Anaerovibrio*	rs320095924	8	3,650,253	SORCS2	0	− 0.364	8.89E−09
*Blautia*	rs331690240	23	14,234,646	ENSSSCG00000050465	0	0.475	2.14E−21
*Bulleidia*	rs340048252	11	69,840,940	NALCN	0	1.086	5.29E−17
*Butyricicoccus*	rs81361511	2	96,004,482	ENSSSCG00000041042	71,388	− 0.882	2.43E−15
*Campylobacter*	rs81472036	18	15,768,346	EXOC4	0	− 0.708	6.09E−07
*Catenibacterium*	rs81323962	3	74,840,151	ETAA1	103,387	− 0.567	8.52E−08
*Clostridium*	rs81251808	4	60,750,431	HNF4G	113,341	− 0.325	3.72E−09
*Collinsella*	rs81430153	11	18,229,756	PHF11	0	0.771	4.32E−07
*Coprococcus*	rs81344777	3	4,420,359	RBAK	2425	− 0.223	1.26E−07
*Desulfovibrio*	rs331690240	23	14,234,646	ENSSSCG00000050465	0	1.052	3.81E−14
*Dorea*	rs81248474	6	8,320,740	ENSSSCG00000042592	0	− 0.199	1.34E−09
*Faecalibacterium*	rs81288412	13	121,545,470	ENSSSCG00000050978	3524	0.237	1.07E−17
*Fibrobacter*	rs343652685	1	174,156,779	ENSSSCG00000047027	156,250	1.092	3.99E−09
*Gemmiger*	rs80980706	17	58,651,427	VAPB	6509	0.269	1.37E−08
*Lachnospira*	rs329723588	15	137,647,629	SCLY	0	− 0.545	1.84E−08
*Lactobacillus*	rs81235044	4	121,603,127	ENSSSCG00000046641	100,786	− 0.743	2.77E−09
*Megasphaera*	rs81371842	3	65,644,946	ENSSSCG00000042171	273,629	− 1.085	1.23E−08
*Mitsuokella*	rs81358080	2	41,479,089	ENSSSCG00000047282	5730	− 1.094	3.68E−08
*Oscillospira*	rs81344398	6	34,452,081	ENSSSCG00000042964	722	− 0.223	4.09E−10
*Parabacteroides*	rs81242782	5	71,843,440	LRRK2	0	− 0.763	2.83E−06
*Peptococcus*	rs81253718	2	43,124,329	ENSSSCG00000045584	48,027	− 0.907	1.44E−09
*Phascolarctobacterium*	rs344728746	15	6,453,282	ENSSSCG00000042287	110,243	− 0.289	2.32E−09
*Prevotella*	rs329143797	8	75,777,330	TRIM2	0	0.178	1.56E−06
*RFN20*	rs81345563	2	44,851,717	ENSSSCG00000033786	38,407	− 0.571	7.63E−12
*Roseburia*	rs81222575	8	19,050,584	ENSSSCG00000041382	5304	− 0.375	1.11E−08
*Ruminococcus*	rs339681838	14	35,548,936	FBXW8	0	0.133	1.26E−07
*Sphaerochaeta*	rs81222575	8	19,050,584	ENSSSCG00000041382	5304	0.776	3.58E−08
*Streptococcus*	rs80787622	11	24,468,975	TNFSF11	0	1.503	2.43E−12
*Succinivibrio*	rs345030123	6	267,415	FANCA	0	0.967	3.82E−09
*Sutterella*	rs322099448	8	74,295,221	RBM46	16,663	0.826	4.34E−12
*Treponema*	rs340048252	11	69,840,940	NALCN	0	− 0.850	2.80E−10
AlphaBACT	rs331027938	7	118,897,310	ENSSSCG00000044157	12,891	0.141	1.10E−07
*Entamoeba*	rs81323991	12	12,377,085	ENSSSCG00000043117	7568	1.175	1.71E−08
*Hypotrichomonas*	rs334181413	18	4,567,215	ENSSSCG00000016426	0	− 1.404	9.73E−09
*Trichomitus*	rs81382396	4	10,874,687	ENSSSCG00000047619	0	1.257	5.98E−07
*Tetratrichomonas*	rs81280147	9	99,713,062	CD36	0	− 0.960	1.57E−08
*Neobalantidium*	rs81323206	6	81,951,114	IFNLR1	37,030	− 1.948	7.05E−09
*Blastocystis*	rs320737267	17	46,864,439	TTPAL	10,153	1.030	2.81E−12
AlphaPROTO	rs80782515	17	28,489,220	RALGAPA2	0	0.373	9.83E−11

**Fig. 1:**
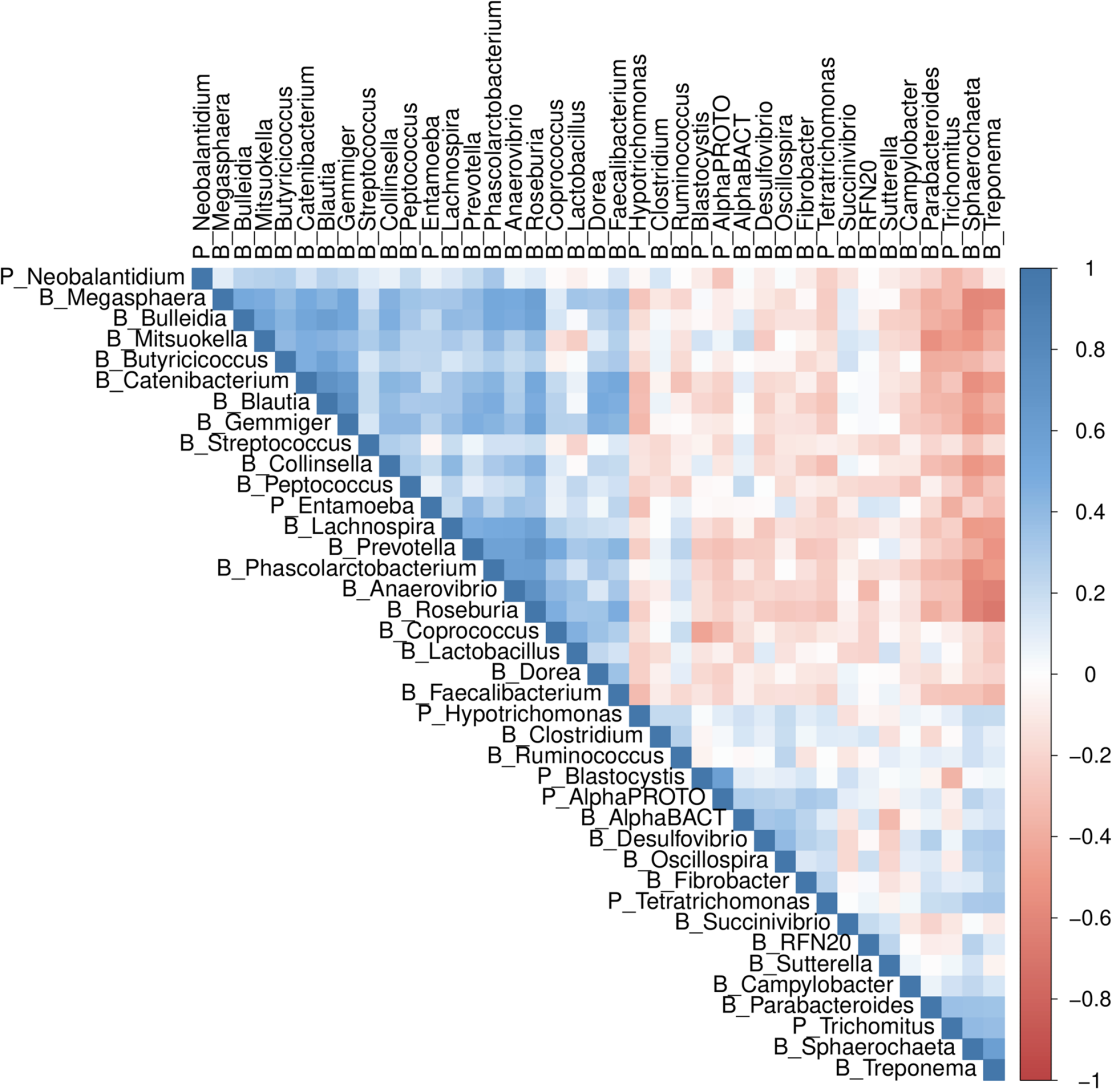
SNP co-association correlation: heatmap of the correlation matrix across 32 bacteria (prefix “B_”) and 7 protist (prefix “P_”) phenotypes based on the 3561 SNP-Genes included in the AWM

The results of GWAS served as the basis for the AWM, the gene co-association network, and the regulatory impact factor approaches. In a first step, for all 39 phenotypes, estimated SNP effects were standardized by dividing them by the standard deviation of all SNP effects. After applying the analytical pipeline described in the “Methods” sections, the resulting AWM was comprised of 3561 SNP anchored to individual genes of which 121 were annotated as TF and 7 microRNA genes. In addition, there were 47 key regulators according to the RIF analyses including 3 microRNA genes. Notably, 10 of the 47 key regulators did not have a significant association (*P* < 0.05) with any of the phenotypes and their relevance would have been overlooked by GWAS alone. For the remaining 3551 genes, the number of associated phenotypes ranged from 1 to 13 and averaged 3.79. A total of 84.08% of the SNPs mapped within genes and the 15.92% were located upstream/downstream of annotated genes. Correlations between microbial traits were calculated using AWM columns (standardized SNP effects across bacteria and protists diversity and abundance) and were visualized as a hierarchical tree cluster, in which strong positive and negative correlations are displayed as proximity and distance, respectively (Fig. 1).

### Gene co-association network linked to microbial phenotypes

Supplementary Fig. 1 shows an overview of the PCIT-inferred gene co-association network for 3561 SNP-Genes included in the AWM procedure, that connected by 738,913 edges of which 374,116 were positive and 364,797 were negative. In the network, node color indicates the phenotype with the strongest association (Supplementary Fig. 1). The network is characterized by a large central module where most of the bacteria vs. protists crosstalk takes place, surrounded by lots of smaller modules mostly phenotype specific. Reflecting the pleiotropic nature of the AWM, while the key phenotypes to capture SNP-Genes were the bacteria and protist alpha diversities, these only represent a minority of the genes: 241 for bacteria alpha diversity (dark blue nodes) and 231 for protist alpha diversity (bright red nodes). In contrast, other bacteria abundance was captured by 2568 genes (light blue nodes), while other protist abundance phenotypes were captured by 521 genes (orange nodes).

### Key regulators in the network

**Fig. 2:**
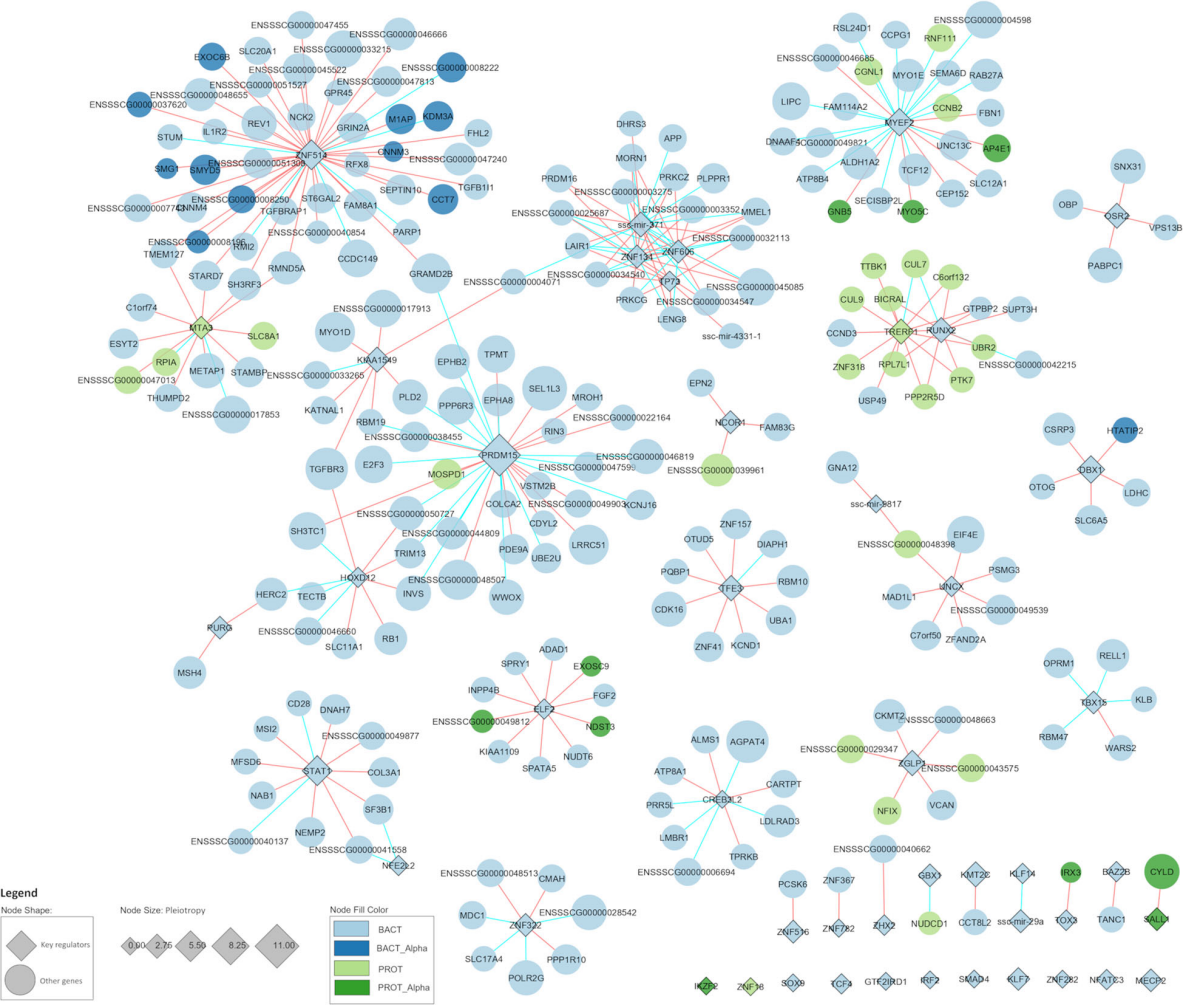
Gene co-association sub-network: PCIT-inferred gene co-association sub-network comprising the 47 key regulators identified by RIF (diamonds) and their first neighbors (ellipses) presenting strong significant correlations (> 0.7). Node color is mapped to the phenotype of the strongest association: dark blue for bacteria alpha diversity (BACT_Alpha), light blue for other bacteria abundance phenotypes (BACT), dark green for protist alpha diversity (PROT_Alpha), and light green for other protist abundance phenotypes (PROT). Pink and cyan edges indicate positive and negative correlations, respectively, and node size indicates the amount of pleiotropy

The RIF analyses identified 47 key regulators among genes selected in the AWM procedure, and these are listed in Table 3, and Fig. 2 resumes the gene co-association sub-network comprising the 47 regulators identified by RIF. Prominent among the 47 key regulators were *PRDM15*, *STAT1*, ssc-mir-371, *SOX9*, and *RUNX2* which gathered 942, 607, 588, 284, and 273 connections, respectively (Table 3). *PRDM15* was associated with 10 traits and was the regulator showing the highest pleotropic value (Table 1). *PRDM15* is a zinc-finger sequence-specific chromatin factor that modulates the transcription of upstream regulators of WNT and MAPK-ERK signaling to safeguard naive pluripotency and regulates the production of Th1-and Th2-type immune response [31]. Similarly, the microRNA ssc-mir-371 has been reported to play an important role in pluripotent regulation in pigs [32]. On the other hand, mice with depleted gut microbiome have been found to develop liver damage and bone loss through the mediation of *SOX9* [33] and *RUNX2* [34], respectively. The signal transducer *STAT1* was associated with five microbial traits (Table 3). *STAT1* has long been associated with immune processes and was recently identified as a potential regulator of vaccine response to porcine reproductive and respiratory syndrome [35]. It should be noted that between 32 and 22.5% of the predicted target genes had at least one TF-binding site for *STAT1* and *PRDM15*, respectively (Supplementary Table 2). Regarding the microRNA ssc-mir-371, a total of 155 binding sites were identified (Supplementary Table 3), comprising a total of 71 different mRNA genes (12% of the initial 588 co-associated genes). The search for background random miRNA-binding sites in the reverse complemented 3′-UTRs gave a total of 115 different binding sites (Supplementary Table 3), comprising 48 different mRNA genes, i.e., the expected number of miRNA 7mer-m8 binding sites for ssc-miR-371 seed was increased by 1.48 folds compared with background random miRNA-mRNA interactions. When we assessed the putative structural consequences of the presence of the rs320008166 (n.59 T > C) mutated allele, a reduction in the minimum free energy (MFE) of the folding of the precursor miRNA hairpin was highlighted. More specifically, the presence of the alternative C allele at position 59th of the precursor region of the miRNA implied the stabilization of a G:U wobble pairing in the wild-type miRNA sequence, introducing a stable canonical Watson-Crick G:C pairing. While the miRNA hairpin carrying the T allele had a MFE = − 35.44 kcal/mol, the presence of the T allele implied an estimated MFE = − 37.74 kcal/mol. Overall, in agreement with AWM original publication [13], here we provide a promoter sequence in silico validation of some of the predicted TF-target genes and also for the first time predicted miRNA target genes.

**Table 3:** List of 47 key regulators revealed by the *regulatory impact factors* (RIF) analyses and their RIF1 and RIF2 scores, standardized association to the bacteria (Alpha_B) and protist (Alpha_P) diversity, phenotype of strongest association, pleiotropy, and number of connections in the PCIT-inferred network

Regulator	RIF1	RIF2	Alpha_B	Alpha_P	Top_Association	Pleio	Conn
PRDM15	0.648	2.806	0.239	0.277	B_Treponema	10	942
HOXD12	1.342	2.536	− 0.667	− 0.624	B_Sphaerochaeta	1	910
ZNF514	0.045	2.607	− 2.938	0.310	B_Sphaerochaeta	5	889
KIAA1549	1.270	2.715	− 1.701	− 1.465	B_RFN20	3	812
KLF7	− 5.625	0.204	− 0.603	1.249	B_Bulleidia	2	807
CREB3L2	− 2.411	0.511	− 0.694	0.818	B_Sphaerochaeta	0	676
TFE3	− 0.127	2.144	1.045	− 0.216	B_Phascolarctobacterium	3	627
TBX15	2.506	1.414	− 0.373	− 1.229	B_Anaerovibrio	2	617
STAT1	− 1.464	2.285	− 1.043	0.370	B_Catenibacterium	5	607
PURG	1.542	2.173	− 1.320	0.338	B_Sphaerochaeta	2	595
ssc-mir-371	− 2.848	1.073	1.181	− 0.690	B_Roseburia	3	588
MTA3	0.390	2.097	− 0.952	− 0.107	P_Hypotrichomonas	2	556
OSR2	− 2.512	− 0.194	0.270	0.005	B_Lachnospira	3	473
DBX1	− 2.047	− 0.129	− 2.207	− 1.577	B_Peptococcus	4	468
UNCX	− 3.085	− 0.194	− 0.051	1.258	B_Desulfovibrio	1	426
MYEF2	− 0.754	− 2.657	1.215	2.430	B_Fibrobacter	4	391
GBX1	− 0.730	− 2.439	− 0.245	− 1.460	B_Campylobacter	2	380
ZNF134	− 2.419	0.128	− 0.572	1.686	B_Lactobacillus	2	370
ZNF606	− 2.419	0.128	− 0.572	1.686	B_Lactobacillus	2	370
SOX9	− 2.524	− 0.404	− 0.498	0.096	B_Anaerovibrio	0	284
KMT2C	− 0.311	− 2.260	− 0.628	− 0.811	B_Catenibacterium	3	281
RUNX2	− 3.631	− 1.878	− 0.206	− 1.776	B_Collinsella	3	273
ELF2	− 1.740	− 2.300	− 0.739	1.688	B_Mitsuokella	1	245
ZNF322	− 2.079	− 0.792	− 0.233	0.809	B_Collinsella	2	229
IRF2	− 0.764	− 2.656	− 0.697	− 1.697	B_Peptococcus	0	209
GTF2IRD1	2.117	0.660	− 1.421	0.132	B_Lachnospira	0	206
ZNF516	2.099	0.150	2.010	0.690	B_Fibrobacter	2	195
NFE2L2	− 2.077	− 0.227	0.705	− 0.051	B_Coprococcus	1	183
NCOR1	− 1.999	− 0.455	− 1.007	1.063	B_Coprococcus	2	165
KLF14	− 2.287	− 2.255	− 0.327	− 1.073	B_Sutterella	1	161
ZGLP1	2.029	− 0.491	0.577	0.729	B_Butyricicoccus	3	161
TCF4	− 2.497	− 1.382	0.280	− 0.333	B_Clostridium	1	150
TP73	− 2.788	− 0.275	0.633	− 1.082	B_Campylobacter	1	146
ZNF782	− 2.831	− 0.500	0.382	− 1.811	B_Desulfovibrio	0	132
SALL1	− 1.133	− 2.716	− 0.833	− 2.125	P_AlphaPROTO	2	131
TOX3	− 2.335	− 2.043	0.365	1.641	B_Lactobacillus	1	129
TRERF1	− 2.359	− 1.043	− 0.286	− 2.005	P_Neobalantidium	3	128
MECP2	− 2.059	− 0.864	0.536	− 0.591	B_Dorea	2	118
SMAD4	− 2.460	− 2.206	0.431	1.586	B_Catenibacterium	0	118
IKZF2	− 1.694	− 2.864	− 0.578	− 1.337	P_AlphaPROTO	0	117
ssc-mir-9817	− 1.841	− 2.175	2.358	2.135	B_RFN20	0	116
ZHX2	− 2.028	− 1.124	0.266	− 1.654	B_Desulfovibrio	1	109
ZNF282	− 1.657	− 2.258	0.954	1.573	B_Ruminococcus	0	85
BAZ2B	− 2.287	− 0.786	− 1.879	0.484	B_Clostridium	1	76
ssc-mir-29a	− 2.067	− 2.748	− 0.568	0.811	B_Lachnospira	2	62
NFATC3	− 2.132	− 2.525	0.270	0.442	B_Desulfovibrio	1	24
ZNF18	− 1.353	− 2.134	− 0.490	− 0.929	P_Neobalantidium	0	18

**Fig. 3:**
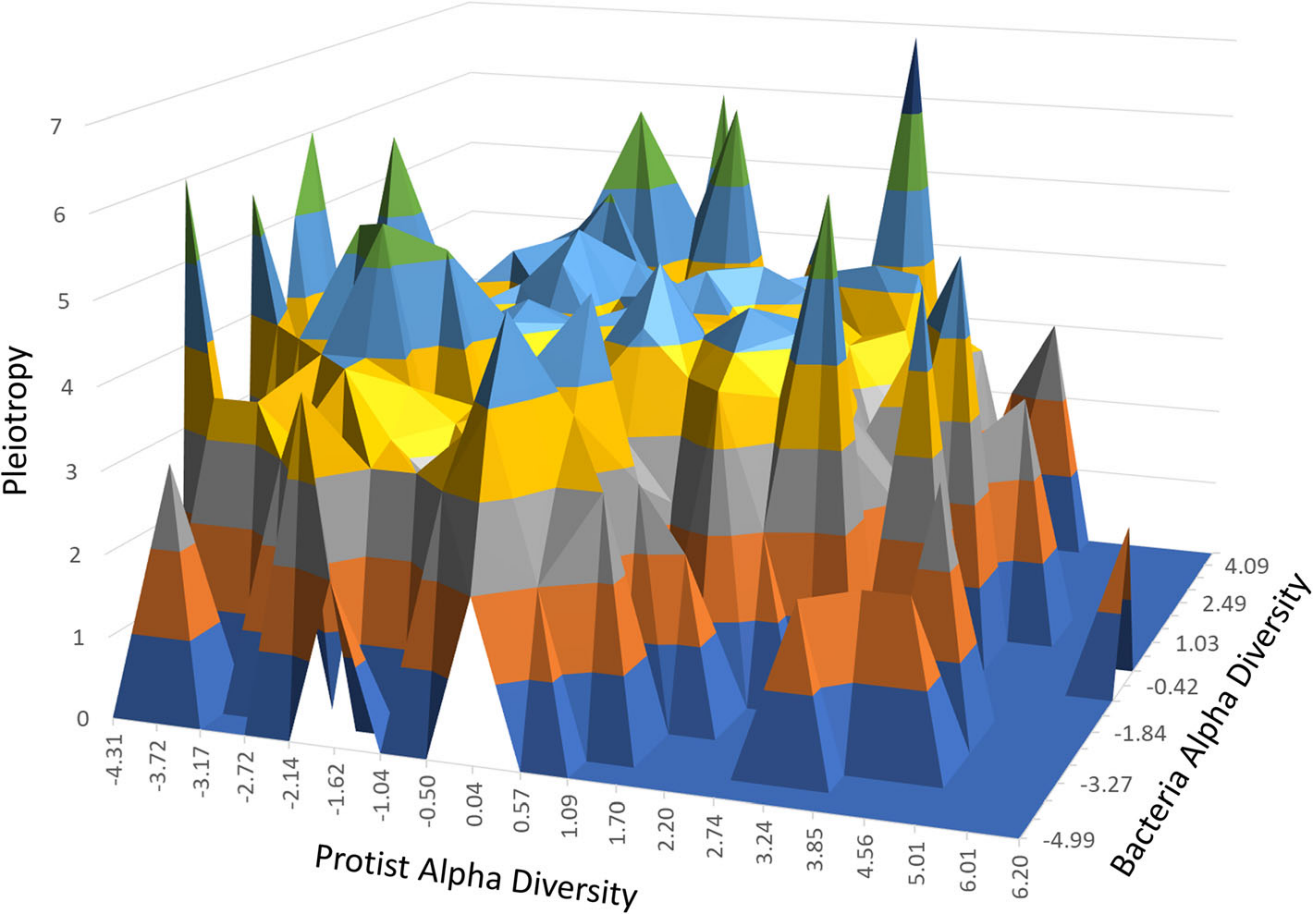
Microbiome diversity and pleiotropy. Surface plot of the 3-way relationship between the SNP association with alpha diversity in protists (width), bacteria (depth) and its pleiotropy (height), measured as the number of significantly associated (adjusted *P* value < 0.05) protist and bacteria genera

A closer inspection of values in Table 3 reveals some fascinating relationships. Pleiotropy (measured by the number of significantly associated phenotypes) and connectivity (number of first neighbors in the co-association network) are significantly correlated (*r* = 0.571; *P* value < 0.0001), suggesting that both metrics are indicators of the pluripotential capacity of the regulators. This finding is of relevance because while pleiotropy was computed from the number of significant phenotypes in the GWAS, the number of connections is a feature of the connectivity in a co-association network. Two vastly different concepts which, when pointing to the same outcome, underscore the relevance of, in this case, regulators. Similarly, while RIF1 and RIF2 scores are moderately correlated (*r* = 0.421; *P* value < 0.01), only RIF2 is significantly correlated with pleiotropy (*r* = 0.471; *P* value < 0.001) and more significantly with connectivity (*r* = 0.806; *P* value < 0.0001). This relationship, to our knowledge, never before documented, indicates the ability of RIF2 scores to prioritize regulators that, in our case study, have an uncanny ability to address the ‘bacteria vs. protists’ crosstalk by differentiating between genes associated with bacterial traits from those associated with protists phenotypes, i.e., the contrast used in developing RIF. To further explore ‘bacteria vs. protists’ crosstalk, Fig. 3 shows the 3-way relationship between a gene’s association with alpha diversity in bacteria, alpha diversity in protists, and its pleiotropy across the 3561 SNP-Genes included in the AWM. While alpha diversities indexes were the key phenotypes used to capture genes for the AWM, a further step aimed at identifying genes with pleiotropic potential (see “Methods”). The plateau of the surface revealed by Fig. 3 indicates that indeed SNP-Genes with near-zero non-significant association with alpha diversity phenotypes were still significantly associated across a large number of other microbial abundance phenotypes which reflect their potential pleiotropic effect.

## Discussions

In this study, we propose a system biology approach to identify candidate genes, regulators, and biological pathways associated with 39 microbial traits. The cluster distribution based on the estimated additive value mirrored the distinct community composition types (enterotype clusters) as well as the known co-occurrence patterns of the pig gut microbiota [4]. For instance, pig gut enterotypes driver taxon *Prevotella* and *Mitsuokella* clustered together and distantly of a second cluster that includes *Treponema* and *Ruminococcus* (Fig. 1). We also noted that butyrate producer genera such as *Faecalibacterium*, *Dorea*, *Blautia*, *Butyrococcus*, and *Coprococcus* tend to cluster closely, which suggest a common directionality of the additive values, and perhaps a common genetic control for this groups of taxa. Notwithstanding the fact that targeting of 16S rRNA variable regions with short-read sequencing platforms cannot achieve the taxonomic resolution at the species level, our results add confidence and suggest the usefulness of the proposed analytical framework to recover key ecological properties of gut microbial ecosystem.

The gene co-association network (Supplementary Fig. 1) revealed the identity of predicted target genes and the higher complexity and polygenic nature of the diversity and composition of the pig gut microbial ecosystem. It is worth noting that literature mining confirms the association between the pig host-genome and the relative abundance of six bacterial genus reported by Crespo et al. 2019 (Supplementary Table 4). Furthermore, our findings also confirm association of 27 of the 68 genes recently reported by [36] as linked with the alpha diversity and the relative abundance of member of the swine gut microbiota (Supplementary Table 5). Remarkably, *PRDM15* was among the genes commonly identify by [36]. As previously mentioned, *PRDM15* was associated with the highest number of traits (Table 3) and the regulator showing the highest pleotropic value, despite differences between studies of genetic background, age, diets, and other environmental factors. Confirmed associations includes 11 of the 17 QTLs reported by Crespo et al. 2019 (Supplementary Table 4), as well as QTLs reported by [36] associated with members of *Clostridium*, *Succinivibrio*, *Bacteroides*, *Prevotella*, *Blautia*, *Turicibacter*, *Treponema*, *Mobiluncus*, and *Oscillibacter* genera. Therefore, our results suggest that in contrast to host genome-microbiota association performed in humans and mouse [37–39], several QTLs reported in swine can be replicated which open the possibility to identify genetic markers and candidate genes that can be incorporated in genetic breeding program to improve microbial traits.

We also noted that the list of target-genes contains a total of 200 candidate genes previously reported as linked to microbial traits in mouse or human studies (Supplementary Table 6). Among them, it is worth highlighting the following: (1) *SLIT3*, reported in the UK Twins Dataset [40] as associated with unclassified *Clostridiaceae* [41], linked to MetaCyc pathways involved in plant-derived steroid degradation, and whose expression is upregulated in colon crypts during the conventionalization of germ-free mice [42]; (2) *SLC39A8*, a gene with a pleiotropic missense variant related with Crohn’s disease and the composition of human gut microbiome [43]; and (3) *NOS1*, for which a pleiotropic association with body fatness and gut microbiota composition in mice had been shown [44]. We also identified other genes that had been shown to be related to β-diversity (*CSMD1*, *ZFAT*, *FRMPD1*, *CLEC16A*, *IL1R2*, *BANK1*, *PRKAG2*, *LHFPL3*, *ST5*, and *NXN*) and gut bacterial abundance (*TOX3*, *NAPG*, *DLEC1*, *COL19A*, *DDM*, and *IFNAR1*) in mice [45–47]. Genes reported by Allison et al. (2019) [48] as differentially expressed in primary human colonic epithelial cells (*NEBL*, *ASAP3*, *ABLIM1*, *CUEDC2*, *PRRC2C*, *DENND1A*, *LAMC1*, *MAL2*, *ITGB1*, *CAST*, *A2ML1*, *IL7R*, *PCDH7*, *NFATC2IP*, *SORCS2*, and *DNM3*) were also found in our study. Furthermore, genes linked to the functional profiles of the human gut ecosystem (*SORCS2*, *LRRC32*, and *ARAP1*) were among the predicted target genes in our network. As previously mentioned, *SORCS2* was reported as linked to a plant-derived steroid degradation pathway. Meanwhile, genetic variants located in *ARAP1* were linked to the bile acid metabolism, and *LRRC32* was associated to the profile of ‘cell–cell signaling’ GO term [41]. It should be noted that many of these genes, including the key regulators, would have been missed by traditional single-trait GWAS which highly the usefulness of the proposed analytical pipeline to identify novels regulators and candidate genes linked with diversity and the composition of pig gut microbial ecosystem.

### Host-genome markers associated with butyrate producing bacteria and piglets body weight

The identification of host-genetic markers linked with the relative abundance of butyrate producer bacteria such as *Faecalibacterium*, *Dorea*, *Blautia*, *Butyrococcus*, and *Coprococcus* (Table 2) prompted us to investigate whether these associations can be expanded in terms of overall pig’s wellbeing, and using the piglets’ body weight as the proxy for health and productivity. Butyrate is an important energy source for intestinal epithelial with anti-inflammatory potential that influences cell differentiation and strengthens the epithelial defense barrier [49, 50]. In fact, the beneficial effect of butyrate on swine growth and intestinal integrity have been documented [51, 52]. Therefore, we focused on the identification of SNPs with pleiotropic effect associated with the relative abundance of butyrate producer bacteria and host performance. After correcting for the systematic effects of sex (2 levels) and batch (7 levels), we found one significant SNP located at 96,004,482 bp of SSC2 (rs81361511) linked with the relative abundance of members of *Butyricicoccus* (*P* = 2.43E− 15) and piglets body weight (*P* = 0.026) (Table 4), as well as two SNPs associated with the relative abundance of *Coprococcus* (rs81344777, *P* = 1.26E− 07) and *Faecalibacterium* (rs81288412, *P* = 1.07E− 17) that were suggestively associated with piglets body weight. It is noteworthy to highlight that in these three cases, the allelic effects were in the same direction: the same allele affects the relative abundance of *Butyricicoccus*, *Coprococcus*, *Faecalibacterium* and piglets body weight. In agreement with [36], our findings suggest the existence of host-genetic variants jointly associated with the relative abundance of beneficial bacterial and host performance. However, larger studies including experimental validations and alternative source of information at host (additional phenotypic traits) and microbial (whole-metagenome, meta-transcriptomics) level are needed to fully characterize the role of the host genome-associated microbial communities in swine production performance, welfare, and health.

**Table 4:** SNPs with pleiotropic effect associated with the relative abundance of butyrate producer bacteria and piglets body weight

SNP	Associated microbial trait	Body weight (60 days)	
		Effect	*P* value
rs81361511	*Butyricicoccus*	1.014	0.026
rs81344777	*Coprococcus*	0.789	0.078
rs81288412	*Faecalibacterium*	− 0.787	0.082

### Host-genome microbial interactions are partially modulated by the host immune system

The functional analysis from the list of regulators reveals overrepresentation of immune-related pathways. As many as 64% (16 out of 25) of the pathways reported as overrepresented by IPA relate to the host immune response (Table 5). Of note, this list includes pathways related with the bidirectional host-microbial crosstalk such as ERK/MAPK signaling [53], Th1 and Th2 activation pathway [54], TGF-β signaling [55], Wnt/β-catenin signaling [56], glucocorticoid receptor signaling [57], VDR/RXR activation [58], IL-22 signaling [59], and aryl hydrocarbon receptor [60].

**Table 5:** List of pathways significantly enriched by the list of regulators

Ingenuity canonical pathways	−log(***P*** value)	Ratio	Genes
Osteoarthritis pathway	3.13	0.019	*RUNX2*,*SMAD4*,*SOX9*,*TCF4*
TGF-β signaling	3.07	0.031	*RUNX2*,*SMAD4*,*TFE3*
Role of osteoblasts, osteoclasts, and chondrocytes in rheumatoid arthritis	3.07	0.018	*NFATC3*,*RUNX2*,*SMAD4*,*TCF4*
Glucocorticoid receptor signaling	2.4	0.012	*NCOR1*,*NFATC3*,*SMAD4*,*STAT1*
VDR/RXR activation	2	0.022	*NCOR1*,*RUNX2*
BMP signaling pathway	1.93	0.024	*RUNX2*,*SMAD4*
Colorectal cancer metastasis signaling	1.89	0.012	*SMAD4*,*STAT1*,*TCF4*
Regulation of IL-2 expression in activated and anergic T lymphocytes	1.89	0.023	*NFATC3*,*SMAD4*
Senescencepathway	1.79	0.011	*ELF2*,*NFATC3*,*SMAD4*
Mouse embryonic stem cell pluripotency	1.77	0.019	*SMAD4*,*TCF4*
Pancreatic adenocarcinoma signaling	1.72	0.018	*SMAD4*,*STAT1*
Neuroinflammation signaling pathway	1.69	0.010	*NFATC3*,*NFE2L2*,*STAT1*
Th1 pathway	1.64	0.016	*NFATC3*,*STAT1*
Human embryonic stem cell pluripotency	1.55	0.015	*SMAD4*,*TCF4*
Aryl hydrocarbon receptor signaling	1.5	0.014	*NFE2L2*,*TP73*
Factors promoting cardiogenesis in vertebrates	1.47	0.013	*SMAD4*,*TCF4*
HOTAIR regulatory pathway	1.41	0.012	*KMT2C*,*TCF4*
Protein kinase A signaling	1.38	0.008	*NFATC3*,*SMAD4*,*TCF4*
Th1 and Th2 activation pathway	1.36	0.013	*NFATC3*,*STAT1*
Wnt/β-catenin signaling	1.35	0.012	*SOX9*,*TCF4*
T cell exhaustion signaling pathway	1.34	0.011	*NFATC3*,*STAT1*
IL-22 signaling	1.34	0.042	*STAT1*
Role of JAK family kinases in IL-6-type cytokine signaling	1.32	0.040	*STAT1*
ERK/MAPK signaling	1.3	0.010	*ELF2*,*STAT1*
Hepatic fibrosis/hepatic stellate cell activation	1.3	0.011	*SMAD4*,*STAT1*

The list of regulator includes other TFs-related with host immune system such as *TFE3*, *NCOR1*, *SMAD4*, *NFE2L2*, *KLF7*, *NFATC3*, *TCF4*, *IRF2*, and *IKZF2*. *TFE3* cooperates with *TFEB* in the regulation of the innate immune response and macrophages activation [61], while *NCOR1* plays an essential role controlling positive and negative selection of thymocytes during T cell development [62]. *SMAD4* regulates IL-2 expression [63] and is essential for T cell proliferation [64]. Interestingly, according to String database [65], experimental data confirm the protein-by-protein interaction between *SMAD4* and previously mentioned regulators *RUNX2*, *SOX9*, *TEF3*, and *NCOR1*. Finally, the list of regulators also includes TFs related to biological process like, inflammatory response (*NFE2L2*, *KLF7*) [66, 67], hematopoiesis (*NFATC3* and *IKZF2*), cell-mediated immune response (*IKZF2*, *IRF2*, *NCOR1*, *NFATC3*, *RUNX2*, *STAT1*, *TCF4*), humoral immune response (*IRF2*, *NFATC3*, *RUNX2*, *STAT1*, *TCF4*), and the modulation of human B-cell differentiation (*KLF14*, *KLF7*, *MTA3*, *STAT1*) [68]. Therefore, in concordance with recent findings in human, mice, and pigs [5, 39, 69], our confirm that host-genome microbial interactions are mainly shaping by the host immune system.

## Conclusions

In the present study, we built and explored a SNP-gene co-association network comprising 3561 genes related to the diversity and abundance of 31 bacterial and 6 commensal protist genera in pigs gut microbiota. Besides identifying genes associated with alpha diversity in both bacteria and protist, our analytical approach takes advantage of the genetic contribution to related microbial traits and revealed genetic variants with pleiotropic effects on pig gut microbiota profile. We also identify SNPs with pleiotropic effect associated with the relative abundance of butyrate producer bacteria (*Faecalibacterium*, *Butyrococcus*, and *Coprococcus*) and host performance. Placing emphasis on regulatory elements, a total of 47 regulators that enriched for immune-related pathways were identified. Among them, five regulators resulted prominent within the network: *PRDM15*, *STAT1*, *ssc-mir-371*, *SOX9*, and *RUNX2*. The list of predicted targets included 200 candidate genes previously reported as associated with microbiota profile in mice and human, such as *SLIT3*, *SLC39A8*, *NOS1*, *IL1R2*, *DAB1*, *TOX3*, *SPP1*, *THSD7B*, *ELF2*, *PIANP*, *A2ML1*, and *IFNAR1*. Taken together, our results highlight the value of the proposed analytical pipeline to exploit pleiotropy and the crosstalk between bacteria and protists as significant contributors to host-microbiome interactions.

## Supplementary Information

**Additional file 1: Supplementary Table 1.** Microbial traits employed in the study.

**Additional file 2: Supplementary Fig. 1.** Overview of the PCIT-inferred gene co-association network for 3561 SNP-Genes included in the AWM procedure.

**Additional file 3: Supplementary Table 2.** List of predicted target genes with at least one TF binding site for STAT1 and PRDM15.

**Additional file 4:** **Supplementary Table 3. **List of predicted target genes with at least one TF binding site for microRNA ssc-mir-371.

**Additional file 5: Supplementary Table 4.** Overlappings QTLs confirmed in our study from the reported by Crespo et al., 2019.

**Additional file 6: Supplementary Table 5.** Overlapping QTLs confirmed in our study from the reported by Bergamaschi et al. 2020.

**Additional file 7: Supplementary Table 6. **List of candidate genes previously reported as linked to microbial traits in mouse or human studies.

## Data Availability

The raw sequencing data employed in this article has been submitted to the NCBI’s sequence read archive (https://www.ncbi.nlm.nih.gov/sra); BioProject: PRJNA608629.

## Abbreviations

QIIME: Quantitative insights into microbial ecology
clr: Centered log ratio transformation
GWAS: Genome-wide association studies
AWM: Association weight matrix
PCIT: Partial Correlation coefficient with Information Theory
RIF: Regulatory impact factors
TF: Transcription factors
miRNA: MicroRNA
