# Supplementary figures and images for: A gene co-association network regulating gut microbial communities in a Duroc pig population
Source: Microbiome. 2021 Feb 21;9:52. doi: 10.1186/s40168-020-00994-8 (PMC7898758; doi:10.1186/s40168-020-00994-8)

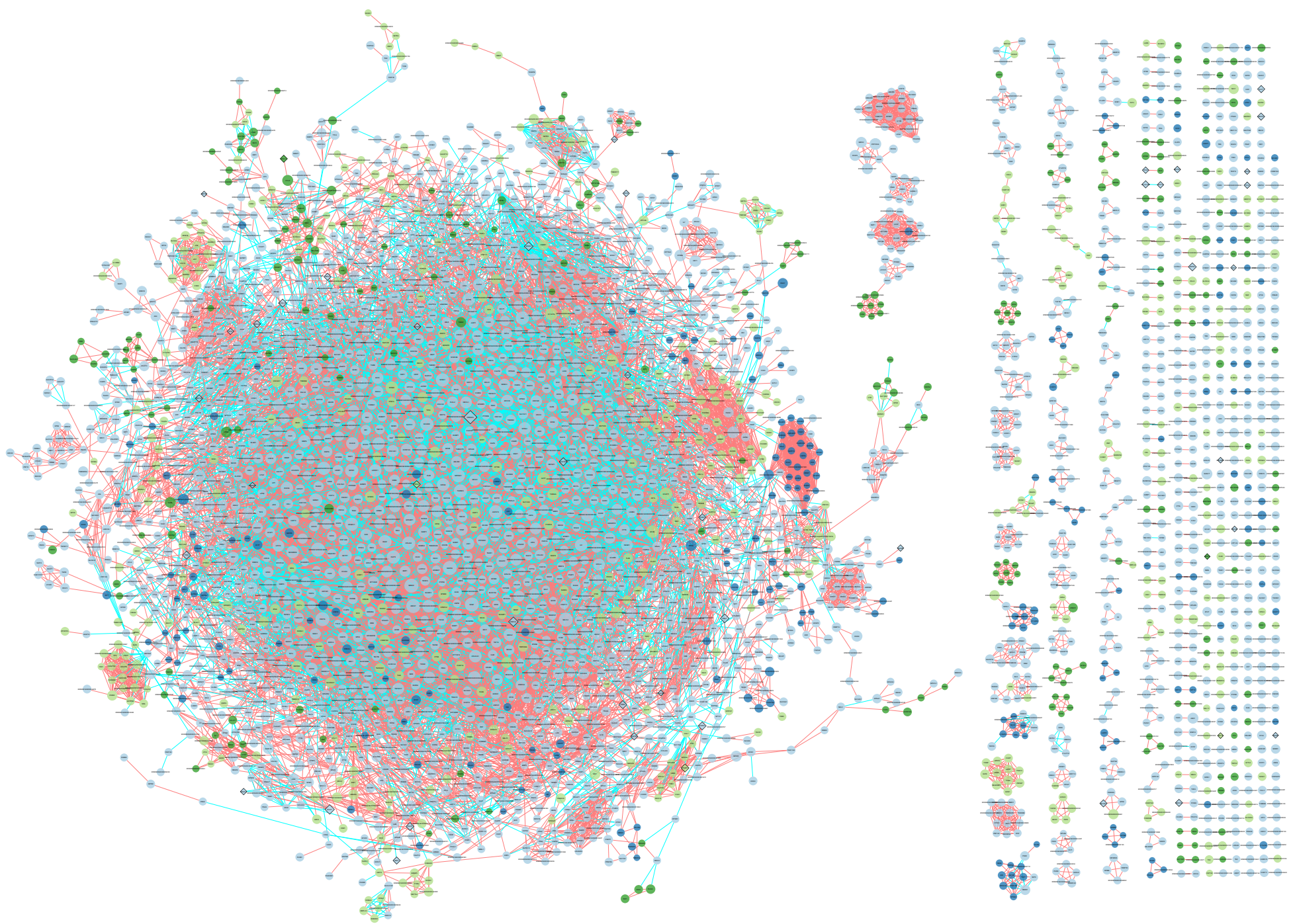

Supplement: Supplementary file 3 — Additional file 2: Supplementary Fig. 1. Overview of the PCIT-inferred gene co-association network for 3561 SNP-Genes included in the AWM procedure. [file 40168_2020_994_MOESM3_ESM.pdf]
